# Blockade of adenosine A_2A_ receptors reverses early spatial memory defects in the APP/PS1 mouse model of Alzheimer’s disease by promoting synaptic plasticity of adult-born granule cells

**DOI:** 10.1186/s13195-023-01337-z

**Published:** 2023-10-30

**Authors:** Qi Ji, Yang Yang, Yun Xiong, Ying-Jie Zhang, Jun Jiang, Li-Ping Zhou, Xiao-Hui Du, Chun-Xiang Wang, Zhi-Ru Zhu

**Affiliations:** 1 Department of Medical Psychology, Army Medical University, Gaotanyan Street 30, Chongqing, 400038 PR China; 2 College of Basic Medicine, Army Medical University, Chongqing, 400038 China; 3 Department of Neurosurgery, The 904Th Hospital of PLA, Medical School of Anhui Medical University, Wuxi, 214044 Jiangsu China

**Keywords:** Alzheimer’s disease, Spatial memory, Adenosine A_2A_ receptors, LTP, Adult born granule cells

## Abstract

**Background:**

The over-activation of adenosine A_2A_ receptors (A_2A_R) is closely implicated in cognitive impairments of Alzheimer's disease (AD). Growing evidence shows that A_2A_R blockade possesses neuroprotective effects on AD. Spatial navigation impairment is an early manifestation of cognitive deficits in AD. However, whether A_2A_R blockade can prevent early impairments in spatial cognitive function and the underlying mechanism is still unclear.

**Methods:**

A transgenic APP/PS1 mouse model of AD amyloidosis was used in this study. Behavioral tests were conducted to observe the protective effects of A_2A_R blockade on early spatial memory deficits in 4-month old APP/PS1 mice. To investigate the underlying synaptic mechanism of the protective effects of A_2A_R blockade, we further examined long-term potentiation (LTP) and network excitation/inhibition balance of dentate gyrus (DG) region, which is relevant to unique synaptic functions of immature adult-born granule cells (abGCs). Subsequently, the protective effects of A_2A_R blockade on dendritic morphology and synaptic plasticity of 6-week-old abGCs was investigated using retrovirus infection and electrophysiological recordings. The molecular mechanisms underlying neuroprotective properties of A_2A_R blockade on the synaptic plasticity of abGCs were further explored using molecular biology methods.

**Results:**

APP/PS1 mice displayed DG-dependent spatial memory deficits at an early stage. Additionally, impaired LTP and an imbalance in network excitation/inhibition were observed in the DG region of APP/PS1 mice, indicating synaptic structural and functional abnormalities of abGCs. A_2A_R was found to be upregulated in the hippocampus of the APP/PS1 mouse model of AD. Treatment with the selective A_2A_R antagonist SCH58261 for three weeks significantly ameliorated spatial memory deficits in APP/PS1 mice and markedly restored LTP and network excitation/inhibition balance in the DG region. Moreover, SCH58261 treatment restored dendritic morphology complexity and enhanced synaptic plasticity of abGCs in APP/PS1 mice. Furthermore, SCH58261 treatment alleviated the impairment of synaptic plasticity in abGCs. It achieved this by remodeling the subunit composition of NMDA receptors and increasing the proportion of NR2B receptors in abGCs of APP/PS1 mice.

**Conclusions:**

Blockade of A_2A_R improves early spatial memory deficits in APP/PS1 mice, possibly by reversing synaptic defects of abGCs. This finding suggests that A_2A_R blockade could be a potential therapy for AD.

**Supplementary Information:**

The online version contains supplementary material available at 10.1186/s13195-023-01337-z.

## Introduction

Alzheimer’s disease (AD) is an increasingly prevalent condition in the context of global aging and has emerged as a leading cause of death among the elderly, imposing a substantial economic burden worldwide. AD is characterized by extracellular deposition of amyloid-β (Aβ), intracellular hyperphosphorylated tau-containing neurofibrillary tangles, synaptic loss and neuronal death [1]. While the deposition of Aβ was previously considered to be the primary cause of AD-related cognitive impairment, recent evidence suggests that cognitive impairment occurs before the appearance of extracellular Aβ-containing senile plaques in the mouse model for AD and patients with mild cognitive impairmen [2–4]. However, the exact pathological mechanisms remain unknown. Therefore, it is crucial to comprehensively explore the neural mechanisms that contribute to early cognitive impairment as they can provide important clues for early detection and intervention in AD.

Spatial memory is a type of cognitive function impaired in the early stages of AD [5]. Spatial navigation impairment is a behavioral biomarker specific to AD-related dementia pathology that can be identified even in the early stages. Patients of amnestic mild cognitive impairment (aMCI) show significant deficits in spatial reference memory [6]. Evidence from various species indicates that the dentate gyrus (DG) of the hippocampus is essential for spatial discrimination behaviors, emphasizing its importance in spatial reference memory [7, 8]. The DG circuit undergoes continuous modifications through the integration of adult-born dentate granule cells. Neural stem cells located in the subgranular zone of the adult hippocampal DG continuously generate newborn granule cells. Adult-born granule cells (abGCs) gradually integrate into the existing circuitry and contribute to the formation and retention of spatial memory [9]. Immature abGCs aged 4–8 weeks display increased excitability and enhanced synaptic plasticity [10, 11], which contribute to the encoding or retrieval stages of DG-dependent spatial discrimination [12]. Additionally, the unique synaptic plasticity exhibited by abGCs enables them to modulate entorhinal cortex inputs to the DG, hippocampal synaptic plasticity, and the activity of local neural circuits in the DG [13–15]. Mice show spatial memory defects after the production of abGCs is reduced by X-rays [16], further suggesting a link between impaired spatial memory function in early AD and abnormal structure and functional alterations of abGCs.

Recent studies have revealed a close association between Adenosine 2A receptors (A_2A_R) and AD-related cognitive impairment [17]. A_2A_R is widely expressed in the cortex and hippocampus and regulates the release of neurotransmitters, neuronal excitability, synaptic plasticity, and glial cell function [18]. AD patients show abnormally elevated levels of A_2A_R in the hippocampus and cortex, with significantly higher expression in those with mild cognitive impairment compared to healthy individuals [19, 20]. Experimental results using APP/PS1 transgenic AD model mice also demonstrated abnormal elevation of A_2A_R in the hippocampus [21]. Remarkably, wild-type mice exhibit severe impairments in spatial discrimination after the activation of hippocampal A_2A_R by optogenetic or agonist agents [22]. Regular consumption of the adenosine receptor blocker caffeine has been shown to decrease the risk of AD in humans [23]. A_2A_R knockout studies have confirmed the involvement of A_2A_R in hippocampal-dependent spatial reference memory impairment in a β-amyloid (Aβ_1-42_)-based model of early AD [24]. A_2A_R blockade by pharmacological inhibition or downregulation driven by shRNA interference in APP/PS1 mice resulted in a significant improvement in spatial memory [21]. Genetic analysis of AD patients has revealed an association between the A_2A_R gene and abnormal hippocampal neurogenesis in early AD [25]. Animal experiments have also shown that A_2A_R regulates neuronal differentiation in the adult hippocampus of mice [26]. Collectively, these experimental pieces of evidence suggest that the blockade of A_2A_R has beneficial effects on AD.

Clinical and experimental evidence suggests that the abnormal upregulation of A_2A_R in the DG may significantly contribute to early spatial memory impairment in AD. However, it remains unclear whether the blockade of A_2A_R can alleviate early spatial memory impairment in APP/PS1 transgenic mice by reversing synaptic defects of immature abGCs. In the present study, we employed behavioral, electrophysiological, and molecular biology techniques to investigate the effect of A_2A_R blockade on spatial memory performance in the early stage of AD. We observed the effect of A_2A_R blockade on the synaptic function of abGCs in the DG and further explored the underlying mechanism.

## Methods

### Animals and drugs

Mice co-expressing familial Alzheimer’s disease (FAD) mutant human PS1ΔE9 and a chimeric mouse-human APP695 harboring the human Aβ domain and mutations (K595N, M596L) linked to Swedish FAD pedigrees (APPswe) have been previously described [27, 28]. Male APP/PS1 mice and their wild-type (WT) littermates at 4 months of age were used in this study. All mice were obtained from the Nanjing Biomedical Research Institute of Nanjing University (Nanjing, China). The mice were housed in plastic cages with ad libitum access to food and water, and the cages were maintained under standard temperature and light conditions (12-h light/dark cycle). Genotypes of the mice were confirmed by PCR using tail tissue DNA. All procedures were performed following the guidelines of the National Institutes of Health Guide for the Care and Use of Laboratory Animals. APP/PS1 and WT mice received intraperitoneal injections of the selective A_2A_R antagonist SCH58261 (0.1 mg/kg, Sigma, St. Louis, MO, USA) at the same time each day for 3 weeks before behavioral tasks and electrophysiological recording. Another group of littermate WT mice received daily injections of an equal volume of the vehicle.

### Behavioral experiments

Spatial reference memory and spatial working memory were measured using the Morris water maze (MWM), novel object location recognition (OLT), and Y-maze tests. The behavioral tasks were conducted during the light phase using 4-month-old APP/PS1 mice and WT mice. Prior to the behavioral tasks, the mice were given a one-week adaptation period to the experimental environment.

The MWM task was performed using a circular pool (diameter: 120 cm, height: 30 cm) filled with opaque water (24 ± 1 °C). The pool was divided into four quadrants, and distal visual cues were placed on the walls. A circular platform (diameter: 10 cm) was positioned in the center of one quadrant and submerged 1.5 cm below the water surface. During the initial phase, mice underwent four trials per day for 5 consecutive days. Each day, mice were tested in all four quadrants in random order with 30-min inter-trial intervals. In each trial, mice were placed in a quadrant and allowed to find the submerged platform within a maximum of 60 s. If they failed to find the platform, mice were guided to it and allowed to sit on it for 20 s. Two probe trials were performed on day 6 with 30-min inter-trial intervals. The platform was removed, and mice were placed in the quadrant opposite to the platform and allowed to freely swim for 60 s. The swimming tracks were recorded using a computer video tracking system and analyzed using Ethovision 11.5 software (Noldus Information Technology). Various parameters, including escape latency, swimming speed, and the time and distance spent in the target quadrant, were analyzed.

The OLT was performed in an open box (30 × 30 × 45 cm^3^) designed according to previous studies [29]. Prior to the test, mice were placed into the empty box to acclimate to the environment for 10 min. After 24 h, mice were placed in the center of the box containing two identical objects and allowed to freely explore the objects for 10 min before being returned to their cages. The following day, one object was moved to the corner opposite the other object. Mice were placed into the box to freely explore for 10 min. Object exploration was defined as sniffing the objects or orienting the nose tip toward the object at a distance of less than 2 cm. The exploration time percentage of one object was calculated by dividing the time spent exploring that object by the total exploration time for both objects. Additionally, the discrimination index (DI) was calculated as follows: (time of novel location—time of familiar location) / total time of novel and familiar location. The time spent in each object location was calculated using Ethovision 11.5 software (Noldus Information Technology). Between the two trials, the box and objects were wiped with ethanol to remove residual odor.

The Y-maze consisted of three equally distributed arms with dimensions of 30 cm long × 6 cm wide × 15 cm high and a 120° angle between the arms. During the training phase, one of the arms (novel arm) was closed. The mice were positioned in the start arm and allowed to explore the remaining two arms (start and other) for 5 min. The test was performed 1 h later, with the door blocking the novel arm removed. The animals were placed again in the start arm and allowed to explore all three arms for 5 min. The percentage of time traveled in each arm and the number of entries into each arm were recorded and analyzed using Ethovision 11.5 software (Noldus Information Technology). The proportion of time spent in the novel arm was calculated as the total time spent in the novel arm divided by the time spent in all the arms during the test session. Spontaneous alternation was determined by consecutive entries into all three arms. The alternation percentage was defined as [number of alternations / (total number of arm entries—2)] * 100%. Between trials, the maze was wiped with ethanol to remove residual odor.

### Immunohistochemistry

After being anesthetized with 4% isoflurane, mice were perfused transcardially with saline and 4% paraformaldehyde (PFA). Brains were removed and fixed with 4% PFA, and then cut into 30 μm thick coronal sections. The sections were treated with 3% H_2_O_2_ for 10 min and incubated overnight at 4 °C with the primary antibodies mouse anti-A_2A_R (1:500, Abcam, Berlin, Germany) or mouse anti-6E10 (1:500, Abcam, Berlin, Germany), diluted in 3% bovine serum albumin (BSA) and 0.3% Triton X-100. Subsequently, the sections were incubated with biotinylated goat anti-mouse secondary antibody (1:1000, Invitrogen, Carlsbad, CA) for 2 h at room temperature. After incubation, the sections were developed using a DAB kit (Vector Laboratories Inc.). The immunohistochemistry was visualized using a microscope (Olympus, Tokyo, Japan).

### Western blotting

Protein of hippocampus was collected using a RIPA lysis buffer (Beyotime, Shanghai, China) according to the instructions. Protein lysates (30 μg) were separated on 8% SDS-PAGE and transferred to PVDF membranes (Millipore, Germany). Membranes were incubated overnight at 4 °C with the primary antibody (mouse anti-A_2A_R, 1:1000, Abcam, Berlin, Germany). Subsequently, membranes were incubated with peroxidase-conjugated secondary antibody (1:1000, Invitrogen, Carlsbad, CA) for 2 h at room temperature. After the incubation, protein bands were visualized using an ECL Substrate (ThermoFisher, Rockfort, IL) and the band intensity was measured for statistical analysis using the ImageJ software.

### Quantitative real-time PCR

Total RNA was extracted from GCs using the RNeasy mini kit (Qiagen) according to the manufacturer’s instructions. Then, cDNAs were generated with random primers using the PrimeScript™ RT reagent kit (Takara), and quantitative real-time PCR (qPCR) was performed using Universal SYBR green Supermix (Toyobo, Osaka, Japan). Each sample was triplicated for analysis. The primer sequences were as follow: GAPDH: F: 5’-ATCCCTCAAAGCTCAGCGTGTC-3’ and R: 5’-GGGTCTTCATTGC GGTGGAGAG-3’; A_2A_R: F: 5’-TGGCTTGGTGACGGGTATG-3’ and R: 5’-CGCAGGT CTTTGTGGAGTTC-3’. The procedures started with an initial denaturation at 95 °C for 10 min followed by 40 cycles of 95 °C for 5 s and annealing temperature of 60 °C for 30 s. The A_2A_R mRNA expression was analyzed using the 2^−△△^Ct method normalized by level of GAPDH.

### Stereotaxic injections

Murine Moloney leukemia retrovirus (Obio Technology, Shanghai, China) containing the GFP reporter gene driven by a CAG promoter was used to label the dividing neurons. Ten-week-old mice without undergoing behavioral tests were anesthetized with 3% isoflurane and placed in a stereotaxic frame (RWD Life Science) in a flat skull orientation. A midline incision was made to expose the skull surface, and two small burr holes were drilled at the injection site. The retroviral vector (1 μL for each position) was injected into the dorsal DG of the mice at a rate of 0.2 μL/min using a high-precision microsyringe Nanoject III (Drummond, Amarican), following these coordinates: anteroposterior: -2 mm; mediolateral: ± 1.5 mm; and ventral: 2.3 mm. The needle was kept in place for 5 min before removal to allow for maximum diffusion of the retrovirus into the tissue. After the injection, mice were housed in cages to recover for 6 weeks before electrophysiological recording or morphometric analysis of transfected neurons.

### Neuronal morphometric analysis

Brains were cut into coronal sections at a thickness of 40 μm using a freezing microtome. Tissue sections that contained the entire hippocampus were sequentially collected in 8 sets of serial slices. For the morphological analysis of abGCs, selected neurons from each genotype were imaged on a Zeiss LSM710 confocal microscope with a 20X objective and a Z-axis step size of 2 μm. To analyze spine density, secondary dendritic branches were selected from abGCs that were positive for GFP after retrovirus injection. Confocal stacks of images were acquired using a 63 × oil immersion objective, 4 × digital zoom, and a Z-axis step size of 0.2 μm. The images were compressed through Z-projections, and morphometric analysis was performed using the Sholl Analysis plugin of the ImageJ software, as previously described [30].

### Hippocampal slice preparation

Mice without undergoing behavioral tests were deeply anesthetized with 4% isoflurane 1.5 month after retroviral transfection. Brains were rapidly removed in ice-cold artificial cerebrospinal fluid (aCSF) containing (in mM): NaCl 125, KCl 2.5, NaHCO_3_ 25, KH_2_PO_4_ 1.25, MgSO_4_ 1.2, CaCl_2_ 2 and dextrose 10. Horizontal 400-μm slices were generated using a vibrating slicer (Vibratome Company, St, USA) and transferred into an equilibration chamber filled with room temperature aCSF, continuously oxygenated with a mixture of 95% O_2_ and 5% CO_2_. The slices were allowed to incubate in the aCSF for at least one hour before electrophysiological recording.

### Electrophysiological recording

#### Field excitatory postsynaptic potentials (fEPSPs) recording

A micropipette recording electrode filled with aCSF (resistance 1–3 MΩ) was positioned in the DG molecular layer of the prepared hippocampal slice for fEPSPs recording. The fEPSPs were evoked by stimulating the medial perforant pathway (PP) using a bipolar stimulating electrode. Input–output curves were obtained using step-up current pulses (0–600 μA, 100 μA steps). The stimulating intensity that evoked 50% of the maximal fEPSPs amplitude was applied at an interpulse interval of 1 min to establish a stable baseline recording for 10 min. Long-term potentiation (LTP) was induced with five episodes of theta-burst stimulation (at 0.1 Hz) consisting of ten stimuli at 100 Hz, repeated ten times at 5 Hz. Subsequently, fEPSPs were continuously recorded for at least 60 min, using the same stimulation as the baseline at an interpulse interval of 1 min. For the network inhibition test, two consecutive pulses were applied. The stimulus intensity was set at a level that evoked a population spike of 1.5–2 mV amplitude of fEPSP. The inhibition/disinhibition at a given interpulse interval was calculated as the relative change in spike amplitude evoked by the second pulse compared to the amplitude evoked by the first pulse. For the network hyperexcitability test, in a model of spontaneous recurrent seizures, the aCSF being perfused was switched to one that lacked Mg^2+^. Population spikes were recorded after 5 min of perfusion in zero-magnesium aCSF. The number of population spikes for the paired-pulse (P1 and P2) was measured for statistical analysis.

#### Excitatory postsynaptic potentials (EPSPs) recording of GCs

Successful whole cell voltage and current clamp recordings were obtained from DG neurons. The recording electrode was filled with intracellular solution composing of (in mM) the following: 145 potassium gluconate, 0.5 EGTA, 2 MgCl2, 5 HEPES, 5 K-ATP, and 0.4 Na-GTP (pH = 7.4). Bicuculline methiodide (10 μM) was added into external superfusate to block GABAergic synaptic transmission. GFP fluorescence signals were visualized under an infrared differential interference microscope and fluorescence microscopy. GFP-positive (GFP +) newborn neurons located in the inner of granule cell layer were selected as abGCs, while GFP-negative (GFP-) neurons in the inner of the granule cell layer were selected as mature granule cells (mGCs). Electrophysiological characteristics for the distinguishing between the abGCs and mGCs were confirmed by the membrane properties, including resting membrane potential (V_rest_), input resistance (R_in_) and membrane capacitance (C_m_). EPSPs recording of GCs were performed in the current-clamp mode at -70 mV. A bipolar stimulating electrode was placed in the medial PP to evoke the EPSP, and the stimulus intensity was adjusted to produce the EPSP amplitude ranging from 3 to 5 mV, repetitively every 1 min. After 10 min of stable baseline recording, a theta burst stimulation was delivered to induce LTP of GCs. Following theta burst stimulation, EPSPs were continuously recorded for 60 min at least.

#### NMDAR-mediated excitatory postsynaptic currents (EPSCs)

NMDAR-mediated EPSCs recording of GCs was performed in the voltage-clamp mode at -60 mV in the presence of the AMPA receptor antagonist CNQX (10 μM) and the GABA receptor antagonist bicuculline (10 μM). The intracellular solution was composed of the following (in mM): 100 Cs-gluconate, 1.7 CsCl, 10 EGTA, 5 MgCl_2_, and 40 HEPES (adjusted to pH 7.4 with CsOH). For the NR2B-sensitive EPSCs, an additional drug, the selective NR2B receptor antagonist ifenprodil (3 μM), was added to the external solution [31]. For the NR2A-sensitive EPSCs, an additional drug, the selective NR2A receptor antagonist NVP-AAM077 ((R)-[(S)-1-(4-bromo-phenyl)-ethylamino]-(2,3-dioxo-1,2,3,4- tetrahydroquinoxalin-5-yl)-methyl]-phosphonic acid) (50 nM), was added to the external solution [32–34]. NR2B- or NR2A-mediated EPSCs were determined by subtracting the current traces in the presence of ifenprodil or NVP-AAM077 from those in the vehicle, respectively. NMDAR-mediated EPSCs were confirmed by application of 50 μM APV (D-2-amino-5-phosphonovaleric acid, NMDAR blockers). Electrophysiological signals were collected using a MultiClamp 700B amplifier (Axon, USA) and acquired using a Digidata 1550 data acquisition system (Molecular Devices). The series resistance was continually monitored throughout the experiment, and only those that changed less than 20% were accepted for data analysis using pCLAMP 10 software (Molecular Devices).

### Single cells RT-PCR assay

The single-cell RT-PCR assay was performed according to a previous study [35]. Briefly, the slices were transferred to the patch-clamp recording chamber and continuously perfused with diethyl pyrocarbonate-treated, DNase- and RNase-free aCSF. GFP + abGCs or GFP- mGCs in the inner granule cell layer were visually identified under an infrared differential interference microscope and fluorescence microscopy. An autoclaved glass pipette was filled with pipette buffer consisting of reverse transcription buffer and then transferred to the headstage on the micromanipulator for patch-clamp recording. Neurons were patched with negative pressure, and their cytoplasm was gently aspirated into the patch pipette. The pipette tip was then broken into a DNase- and RNase-free PCR tube containing reverse transcription components and immediately snap-frozen. A sample of the bath solution from the vicinity of the neuron was collected to replace the cellular template as a negative control. The first-strand cDNA was synthesized using the High Capacity cDNA Reverse Transcription kit (Invitrogen, Carlsbad, CA, United States), and the target genes were detected by quantitative real-time PCR amplification as previously described. The primer sequences were as follows: NR2A: F: CAACGAAGGGATGAATGTGA, R: ACAAAGGGCACGGAGAAGT; NR2B: F: TGCTACAACACCCACGAGAA; R: CTCCTCCAAGGTAAC GATGC. All PCRs were performed following procedures designed to minimize the chances of cross-contamination.

### Statistical analysis

All data were presented as Mean ± SEM, and statistical analysis was performed using SPSS 21 (Armonk, NY, USA). The statistical analysis for repeated measures data used the one-way repeated-measures ANOVA, including the escape latency and swimming speed in the MWM, input–output curves in electrophysiological recording, and Sholl analysis. Unless otherwise stated, comparisons of nonparametric data were performed using the Mann–Whitney test, and comparisons of parametric data were performed using Student’s t-test (paired or unpaired) or one-way ANOVA followed by Sidak’s post-hoc test. A *p*-value less than 0.05 was considered statistically significant.

## Results

### Over expression of A_2A_R in the hippocampus of APP/PS1 mice

**Fig. 1:**
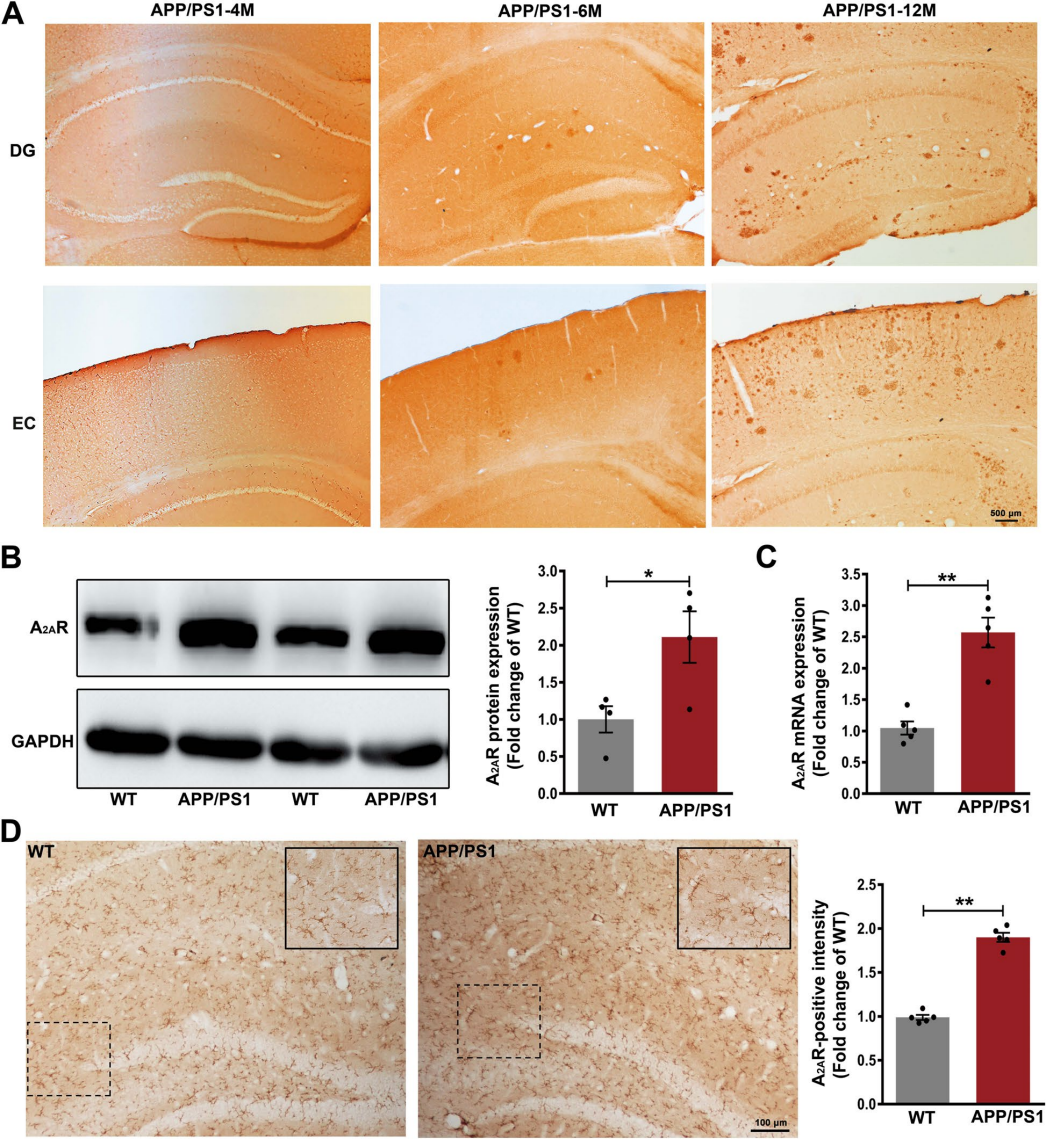
A_2A_R Overexpression in the Hippocampus of APP/PS1 Mice. **A** Representative images of Aβ plaques in the dentate gyrus (DG) and entorhinal cortex (EC) from 4, 6, and 12-month-old APP/PS1 mice. Prominent Aβ plaques were observed in 6 and 12-month-old APP/PS1 mice, but not in 4-month-old APP/PS1 mice. Scale bar = 500 μm. **B** Western blots showed a significant upregulation in protein expression of A_2A_R in the hippocampus of APP/PS1 mice compared to wild-type (WT) mice, *n* = 4 animals per group. **C** mRNA levels of A_2A_R were significantly increased in the hippocampus of APP/PS1 mice compared to WT mice, *n* = 5 animals per group. **D** Representative images of A_2A_R immunohistochemical staining in the DG of mice. Scale bar = 100 μm. Inserts show locally enlarged images of indicated areas by the dashed line boxes, *n* = 5 animals per group. Data are presented as the mean ± SEM, *p < 0.05 and **p < 0.01 by Student’s t-test

We initially examined the presence of amyloid deposits and expression of A_2A_R in APP/PS1 transgenic mice in comparison to their WT littermates. No amyloid deposits were observed in the brains of either 4-month-old APP/PS1 mice or WT mice at any age (date not shown), while they were clearly present in the hippocampus and cortex of 6 and 12-month-old APP/PS1 mice. The severity of amyloid deposits increased with age in APP/PS1 mice (Fig. 1A). Next, the expression of A_2A_R was quantified by western immunoblot analysis, which revealed significantly increased expression of A_2A_R in the hippocampus of 4-month-old APP/PS1 mice compared to WT mice (*p* < 0.05, Fig. 1B). These results were further confirmed by RT-PCR (*p* < 0.01, Fig. 1C). The A_2A_R positive immunoreactivity was also significantly greater in APP/PS1 mice than that in WT mice (*p* < 0.01, Fig. 1D).

### Blockade of A_2A_R ameliorated spatial memory impairment in APP/PS1 mice

**Fig. 2:**
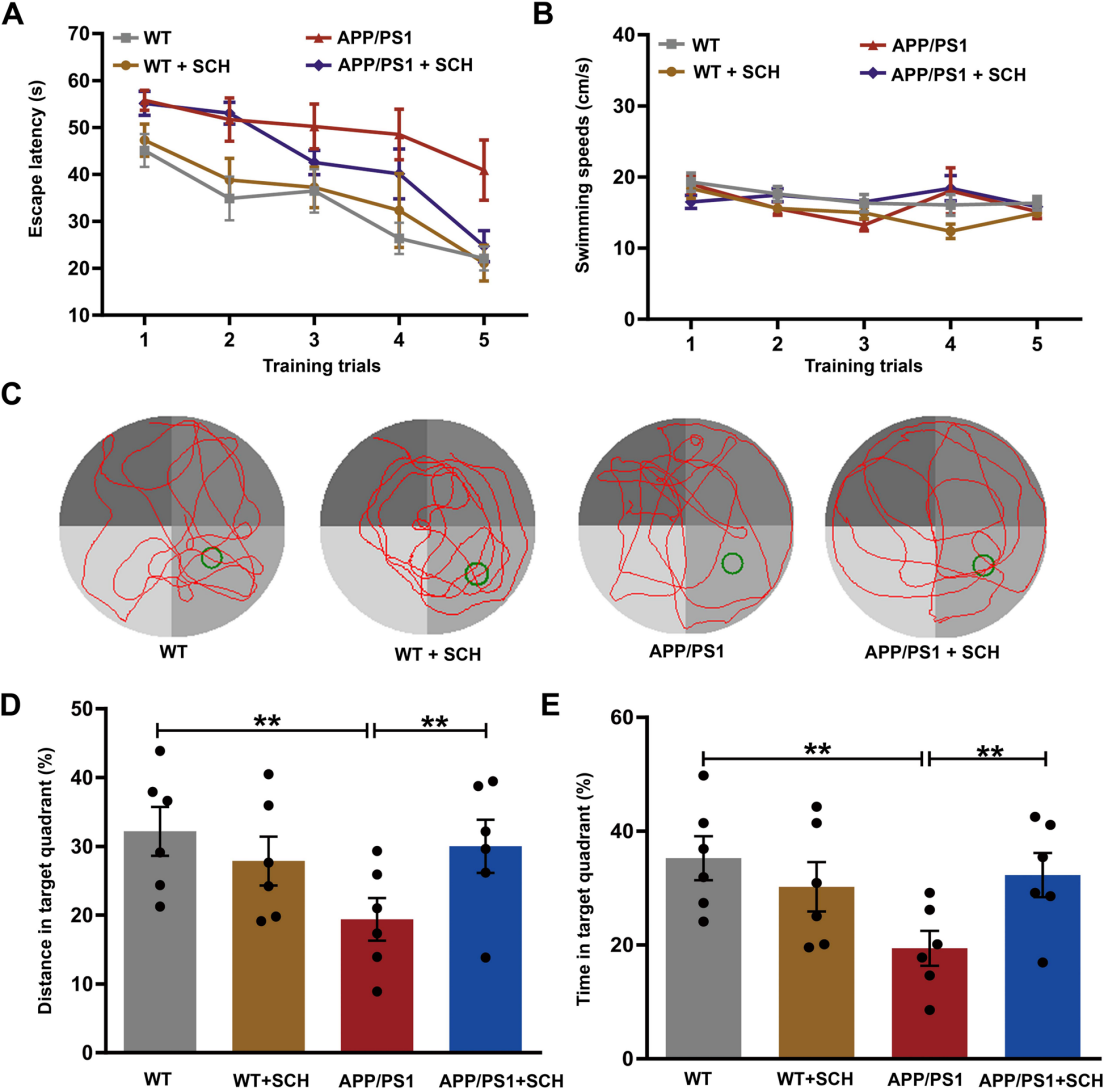
Blockade of A_2A_R Ameliorated Spatial Memory Impairment in APP/PS1 Mice during Morris Water Maze Testing. **A** Spatial discrimination of mice was assessed using MWM tests. Mice exhibited progressively reduced escape latencies in searching for the platform during acquisition training. The escape latencies were significantly longer in the APP/PS1 group than in the WT group, while treatment with SCH58261 decreased the escape latency of APP/PS1 mice. **B** No significant differences in swimming speed were observed between the groups. **C** Representative swimming paths of mice while searching for the hidden platform in probe trials. **D**-**E** Analysis of variance (ANOVA) showed lower percentage of time and distance spent in the target quadrant in APP/PS1 mice compared to WT mice. However, this difference between genotypes was abolished by SCH58261 treatment. Data are presented as the mean ± SEM, *n* = 6 animals per group, *p < 0.05, **p < 0.01 by repeated-measures ANOVA or one-way ANOVA followed by Sidak's post-hoc test

The effect of A_2A_R blockade on the protein expression of A_2A_R in the hippocampus was firstly detected, showing that treatment with SCH58261 had no effect on A_2A_R expression in WT and APP/PS1 mice (Supplementary Fig. 1). Further, The protective effect of SCH58261 on spatial memory impairment in APP/PS1 mice was determined through behavioral testing. Using the Morris water maze, we observed that compared to WT mice, APP/PS1 mice displayed longer platform escape latencies (*p* < 0.01, Fig. 2A), indicating spatial learning and memory defects. However, treatment with SCH58261 significantly prevented spatial learning and memory impairments in APP/PS1 mice (*p* < 0.01, Fig. 2A). SCH58261 alone did not have a significant effect on the escape latency (*p* > 0.05, Fig. 2A). No significant differences in swimming speed were found between the groups (*p* > 0.05, Fig. 2B). During spatial probe trials, APP/PS1 mice showed a significantly lower percentage of time and distance spent in the target quadrant compared to WT littermates (*p* < 0.01 and *p* < 0.01 respectively, Fig. 2C-E). However, this difference between genotypes was eliminated by SCH58261 treatment (*p* < 0.01 and *p* < 0.01 respectively, Fig. 2C-E), indicating that A_2A_R blockade improved the spatial learning and memory of APP/PS1 mice. SCH58261 alone did not have a significant effect on the percentages of distance and time spent (*p* > 0.05, Fig. 2C-E).

**Fig. 3:**
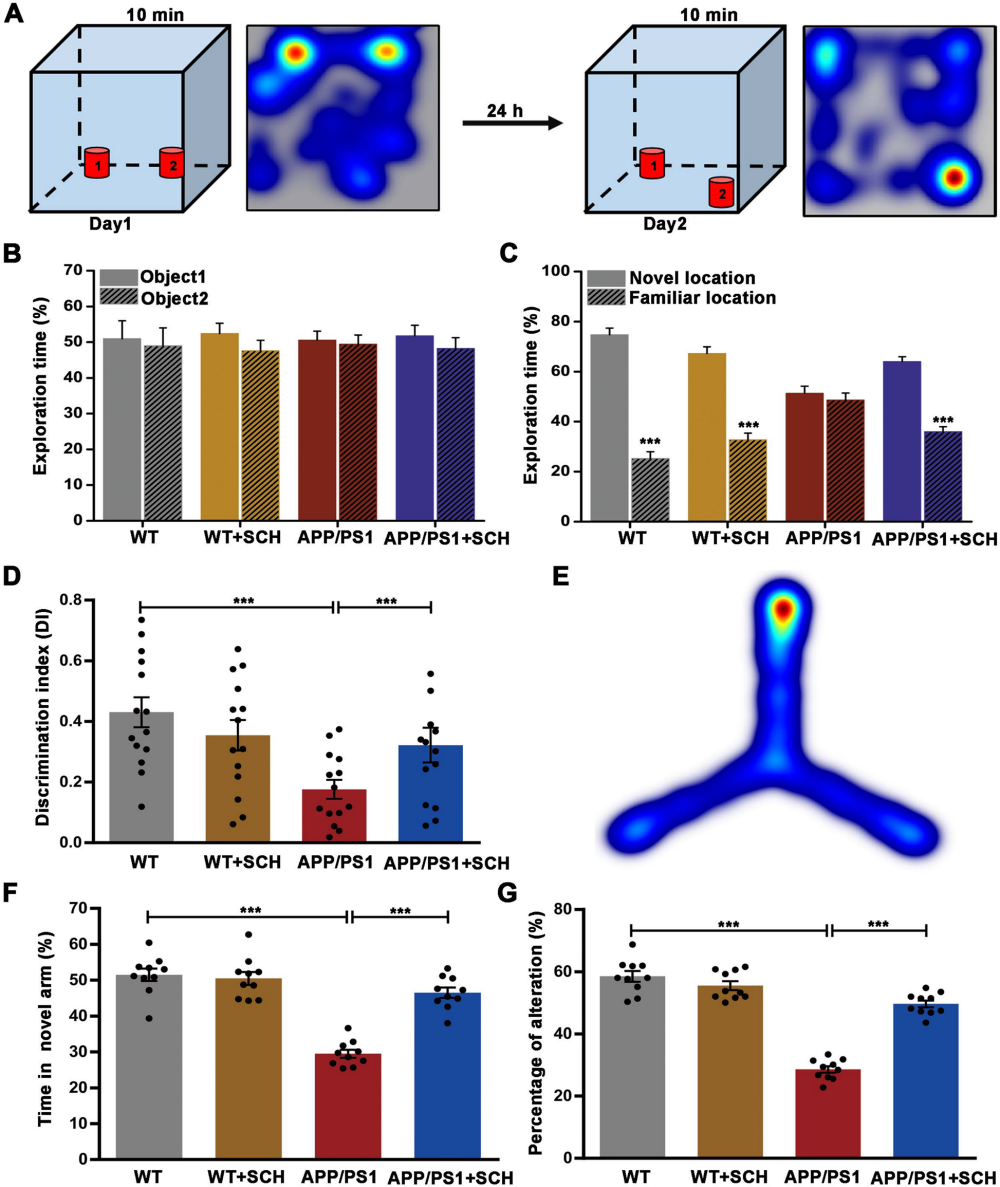
Blockade of A_2A_R Ameliorated Spatial Memory Impairment in APP/PS1 Mice during OLT and Y Maze. **A** Simplified scheme of the OLT test in mice, *n* = 14 animals per group. **B** No significant differences in the time exploring each of two identical objects were observed between the groups. **C** In the test session, WT mice showed significant preference for the novel location. APP/PS1 mice showed no object location preference, while SCH58261 treatment reversed location preference of APP/PS1 mice. **D** The summary bar graph illustrates that compared to WT mice, APP/PS1 mice exhibited a lower discrimination index. Notably, treatment with SCH58261 increased the discrimination index in APP/PS1 mice. **E** Heat map of Y Maze Test, *n* = 10 animals per group. **F**-**G** Y maze assessment found that APP/PS1 mice spent less time in the novel arm (**F**) and had a significantly lower percentage of spontaneous alternation (**G**). The SCH58261 treatment reversed this in APP/PS1 mice. Data are presented as the mean ± SEM, ***p < 0.001 by Student’s t-test or one-way ANOVA followed by Sidak’s post-hoc test

Then, the novel object location test (OLT) was carried out to evaluate location recognition memory (Fig. 3A). No significant difference was found in the time spent exploring two identical objects during the familiarization trial (*p* > 0.05, Fig. 3B). In the test session, WT mice spent significantly more time exploring the novel location than the familiar location (74.73% vs. 25.27%, *p* < 0.001, Fig. 3C), whereas the time spent exploring the two objects was similar in APP/PS1 mice (51.35% vs. 48.65%, *p* > 0.05, Fig. 3C). Furthermore, the discrimination index was significantly lower in APP/PS1 mice compared to WT mice (*p* < 0.001, Fig. 3D). Notably, SCH58261 treatment significantly prolonged the time spent exploring the novel location and increased the discrimination index in APP/PS1 mice (*p* < 0.001, Fig. 3C-D), indicating that A_2A_R blockade improved novel position recognition in APP/PS1 mice.

Subsequently, the Y-maze test was performed to assess hippocampus-dependent spatial working memory (Fig. 3E). In comparison to WT mice, APP/PS1 mice spent less time in the novel arm and had a significantly lower percentage of spontaneous alternation (*p* < 0.001 and *p* < 0.001, Fig. 3F, G). However, SCH58261 treatment reversed this in APP/PS1 mice (*p* < 0.001 and *p* < 0.001, Fig. 3F, G), indicating that the A_2A_R blockade improved spatial working memory in APP/PS1 mice. Collectively, these results suggested that blockade of A_2A_R could ameliorate spatial memory impairment in APP/PS1 mice.

### Blockade of A_2A_R ameliorated LTP impairment in the DG of APP/PS1 mice

**Fig. 4:**
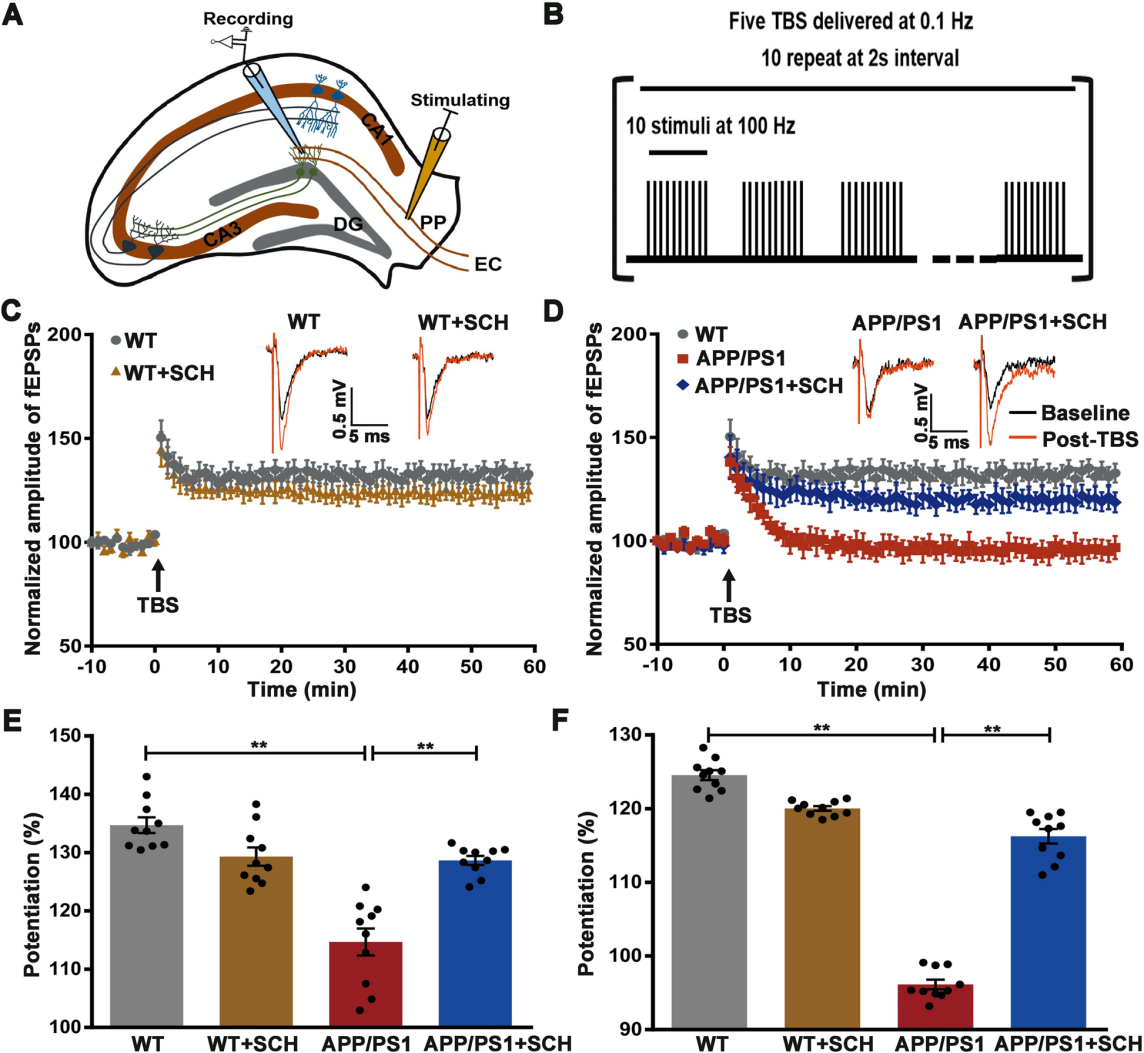
Blockade of A_2A_R Recovered LTP of the DG Region in APP/PS1 Mice. **A** Schematic illustration of field excitatory postsynaptic potential (fEPSP) recordings in the DG. **B** Schematic illustration of theta-burst stimulation for the induction of LTP. **C** fEPSPs of the DG region were recorded in acute mouse hippocampal slices. The mean amplitude of fEPSPs, expressed as a percentage of the baseline level, is plotted for the WT group and the WT + SCH58261 group. It shows the 10 min of baseline recording and 60 min of post-TBS (arrow) recording. Responses were evoked and collected at a rate of 1/60 s. Insets represent typical fEPSPs recorded before (black) and 50 min after (red) TBS. Scale bar = 5 ms and 0.5 mV. **D** Summary of experiments showing LTP recorded from the DG region in slices from WT mice, APP/PS1 mice, and SCH58261-treated APP/PS1 mice. **E**–**F** The average amplitude of fEPSPs during the first 10 min (**E**) and the last 10 min (**F**) post-TBS was significantly decreased in APP/PS1 mice. However, this decrease was significantly reversed by SCH58261 treatment. Data are presented as the mean ± SEM, *n* = 10 animals per group, **p < 0.01 by one-way ANOVA followed by Sidak's post-hoc test

To investigate the synaptic mechanism underlying impairments in spatial learning and memory, we examined LTP in the DG region using previous methods [36]. The field excitatory postsynaptic potential (fEPSP) of the DG region was evoked by stimulating the perforant pathway in acute hippocampal slices (Fig. 4A). For LTP induction, we applied five episodes of theta-burst stimulation at 0.1 Hz (TBS, 10 stimuli at 100 Hz, repeated ten times at 5 Hz, Fig. 4B), a robust protocol for LTP induction. TBS was delivered to brain slices from both WT and APP/PS1 mice following a 10-min stable baseline at 1-min intervals. The fEPSP amplitude was normalized to the average fEPSP amplitude during the baseline (Fig. 4C-D). Typical fEPSP traces from different groups are shown in the upper row. The average fEPSP amplitude during the first 10 min after TBS (0–10 min) and the average fEPSP amplitude during the last 10 min after TBS (50–60 min) were compared to assess LTP induction and maintenance, respectively (Fig. 4E-F). In WT mice, the amplitude of fEPSP increased immediately after TBS and then plateaued, indicating successful LTP induction and maintenance (Fig. 4C). However, the average fEPSP amplitude was decreased in APP/PS1 mice compared to WT mice (the first 10 min: WT 134.69 ± 1.33% vs APP/PS1 114.66 ± 2.31%,* p* < 0.01, Fig. 4E; the last 10 min: WT 124.67 ± 0.62% vs APP/PS1 96.42 ± 0.65%, *p* < 0.01, Fig. 4F), suggesting impaired LTP induction and maintenance in APP/PS1 mice. SCH58261 treatment recovered the average fEPSP amplitudes in APP/PS1 mice (the first 10 min: 128.65 ± 0.77%, *p* < 0.01, Fig. 4E; the last 10 min: 116.09 ± 0.91%, *p* < 0.01, Fig. 4F), indicating that A_2A_R inhibition by SCH58261 prevents fEPSP impairment in APP/PS1 mice. SCH58261 alone had no significant effect on LTP induction and maintenance (*p* > 0.05, Fig. 4F). Altogether, these results suggest that blockade of A_2A_R ameliorated the impairment of LTP in APP/PS1 mice.

### Blockade of A_2A_R recovered network inhibition in the DG of APP/PS1 mice

**Fig. 5:**
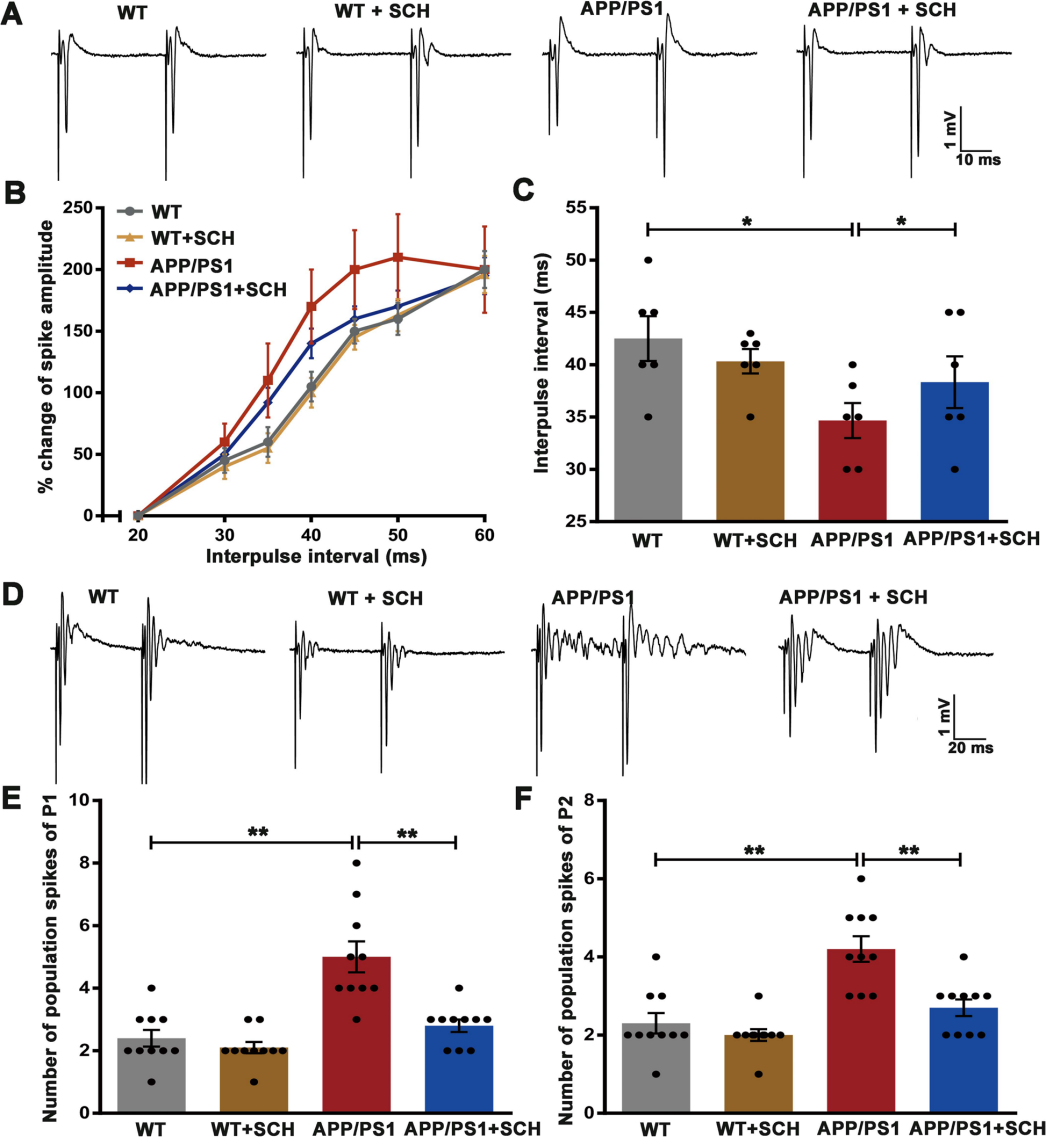
Blockade of A_2A_R Enhanced DG Network Inhibition and Inhibited Neuronal Hyperexcitability. **A** Typical paired pulses responses at 50 ms interpuls interval evoked by stimulation of MPP in slices from different groups mice in normal aCSF. Scale bar = 10 ms and 1 mV, *n* = 6 animals per group. **B** Paired-pulse inhibition (PPI) of the population spike was examined in different groups of mice at a stimulation intensity which evoked a population spike of 1.5–2 mV amplitude. The PPI curve of APP/PS1 mice was shifted towards weaker inhibition. Treatment with SCH58261 shifted the paired-pulse inhibition curve of APP/PS1 mice toward control level. **C** Paired-pulse inhibition curves were fitted to the Boltzmann function and a hypothetical interpulse interval (IPI), at which equal amplitude of the first and the second population spike would be detected, was determined and compared between groups. The IPI was significantly shortened in APP/PS1 group, as shown in the bar graph. Treatment of SCH58261 prolonged the IPI of APP/PS1 mice. **D** Representative field potentials recorded in the granule cell layer of DG in response to paired electrical stimulus applied to MPP in Mg^2+^- free aCSF. Scale bar = 20 ms and 1 mV, *n* = 10 animals per group. **E**–**F** The number of population spikes overriding the fEPSPs in pulse1 (P1, E) and pulse2 (P2, F). The number of both P1 and P2 were significantly increased in APP/PS1 mice compared to WT mice. Treatment of SCH58261 decrease population spike frequency of APP/PS1mice. Data are presented as the mean ± SEM, *p < 0.05, **p < 0.01 by one-way ANOVA followed by Sidak’s post-hoc test

In our recent study, we found that an excitation/inhibition imbalance contributes to LTP impairment in the DG region [37]. The modulation of population spike amplitude during paired-pulse stimulation serves as an indicator of network inhibition [38]. To test whether the protective effects of A_2A_R blockade on DG LTP in APP/PS1 mice involve modulation of the network balance, we analyzed changes in population spike amplitude following paired-pulse stimulation (Fig. 5A). Using a stimulation intensity that elicited a population spike amplitude of 1.5–2 mV, the network inhibition at a given interpulse interval was calculated as the relative change in the amplitude of the population spike evoked by the second pulse compared to the first pulse. The paired-pulse inhibition (PPI) curve of APP/PS1 mice showed weaker inhibition compared to WT mice (Fig. 5B). To quantify the shift, we fitted the PPI curves to the Boltzmann function and calculated the mean interpulse interval (IPI) at which equal amplitudes of the first and second population spike occurred. The mean IPI was significantly lower in the APP/PS1 group compared to the WT group (33.17 ± 1.35 ms vs 42.50 ± 2.14 ms, *p* < 0.05, Fig. 5C), indicating reduced network inhibition in APP/PS1 mice. Interestingly, SCH58261 treatment reversed this difference between genotypes (39.17 ± 2.01 ms, *p* < 0.05, Fig. 5C), suggesting that blockade of A_2A_R increased network inhibition in the DG region.

To further explore the role of A_2A_R blockade in the network excitation/inhibition imbalance of the DG region, we compared the effect of removing extracellular Mg^2+^ ions on slices from WT and APP/PS1 mice. Slices from both WT and APP/PS1 mice exhibited hyperexcitability within 5 min after exposure to Mg^2+^-free aCSF with a marked increase in the number of population spikes, which was exacerbated in the APP/PS1 mice (Fig. 5D). The APP/PS1 mice showed a significant increase in the number of population spikes following the first and second stimuli compared to WT mice (P1: 5.00 ± 0.49 vs 2.40 ± 0.27 ms, *p* < 0.01, Fig. 5E; P2: 4.20 ± 0.33 vs 2.30 ± 0.26, *p* < 0.01, Fig. 5F). Treatment with SCH58261 significantly decreased population spike frequency in APP/PS1 mice (P1: 2.80 ± 0.20, *p* < 0.01, Fig. 5E; P2: 2.70 ± 0.21, *p* < 0.01, Fig. 5F). These results suggest that blockade of A_2A_R increases network inhibition and recovers the network excitation/inhibition balance of the DG region.

### A_2A_R blockade reversed synaptic defects of abGCs neurons in APP/PS1 mice

**Fig. 6:**
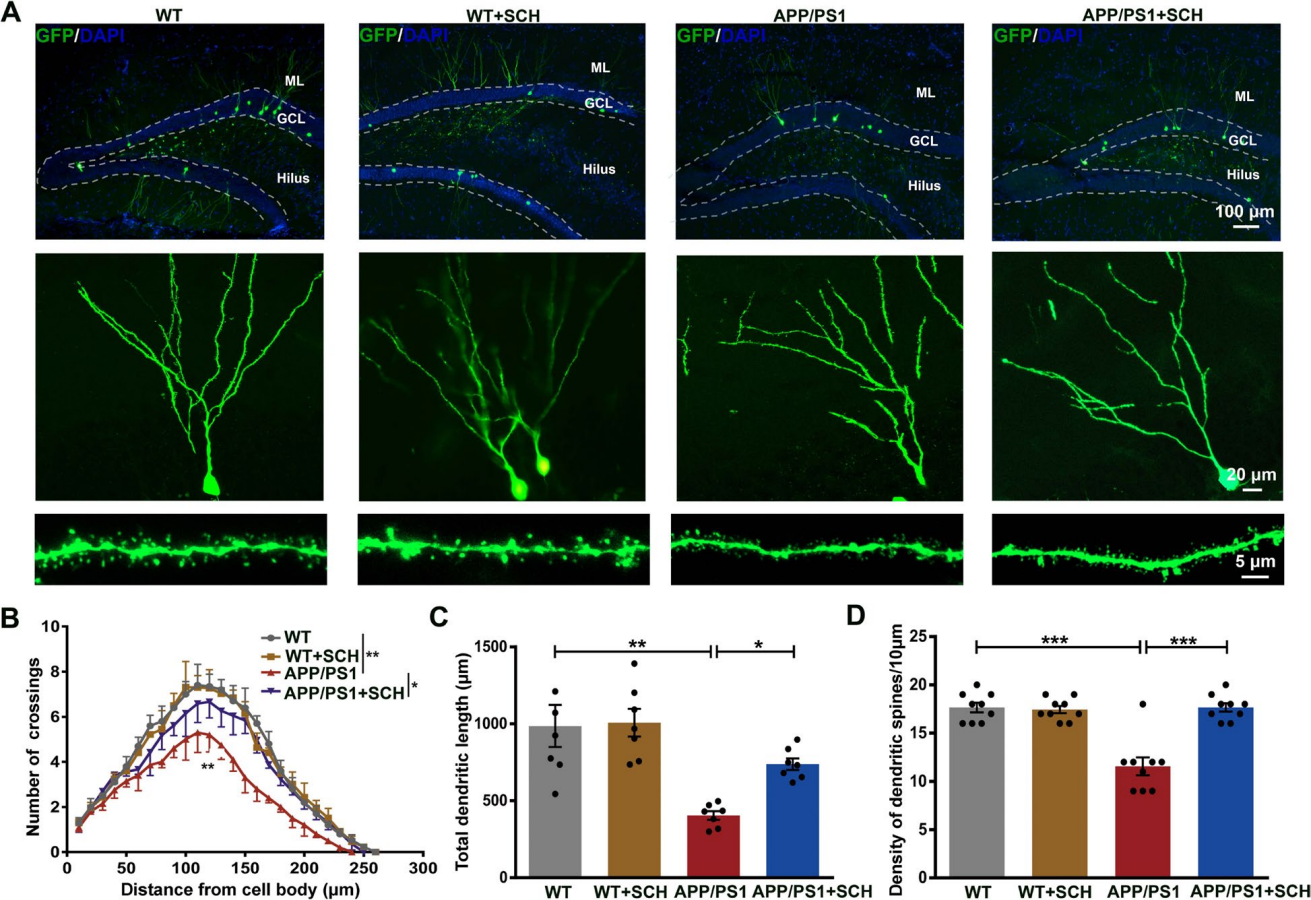
Blockade of A_2A_R Reversed the Alterations in Dendritic Morphology of abGCs in APP/PS1 Mice. **A** Representative images of 1.5-month post-injection (mpi) GFP + abGCs from different groups of mice. Upper: low magnification image showing the location of GFP + abGCs, scale bar = 100 μm. White dotted lines were added to mark the boundary line between the granule cell layer (GCL), molecular layer (ML), and hilus. Middle: representative maximal projection for dendritic morphological analysis, scale bar = 20 μm. Lower: high-power magnification image showing examples of dendritic spines, scale bar = 5 μm. **B** Quantification of dendritic arborization using Sholl analysis showed that abGCs of APP/PS1 mice displayed a significant decrease in dendritic branching compared to WT mice, which was reversed by SCH58261 treatment, *n* = 7 neurons per group, with each neuron from an individual animal. **C** Histograms showing a decrease in total dendritic length for abGCs of APP/PS1 mice compared to WT mice. However, treatment with SCH58261 partially reversed the decrease in the total dendritic length for abGCs of APP/PS1 mice. **D** Histograms showing a significant reduction in dendritic spine density in abGCs of APP/PS1 mice compared to WT mice, which was reversed by SCH58261 treatment, *n* = 9 neurons per group, with each neuron from an individual animal. Data are presented as the mean ± SEM, *p < 0.05, **p < 0.01, ***p < 0.001 by repeated-measures ANOVA or one-way ANOVA followed by Sidak's post-hoc test

It has been demonstrated that modulation of adult neurogenesis has a profound impact on hippocampal synaptic plasticity, network activity in the DG, and spatial memory [14, 15, 39]. AbGCs play a crucial role in integrating into the existing circuit, forming synapses with matured granule cells (mGCs) and interneurons [40]. The unique synaptic plasticity exhibited by abGCs enables them to modulate hippocampal synaptic plasticity and the activity of local neural circuits in the DG [13–15]. So, SCH58261 may regulate LTP and the E/I balance in the DG of APP/PS1 mice by modulating the synaptic function of abGCs. To investigate whether the protective effects of A_2A_R blockade in APP/PS1 mice were associated with rescuing synaptic defects of abGCs, we observed the morphology of abGCs 1.5 month after retrovirus injection. As shown in Fig. 6A, most of the 1.5-month post-injection (mpi) GFP + granule cells are located at the hilar border of the granule cell layer, with their apical dendrites extending toward the molecular layer. Quantification of dendritic arbor complexity by Sholl analysis revealed marked alterations in the dendritic branching of abGCs in APP/PS1 mice. AbGCs from APP/PS1 mice showed a significant decrease in dendritic branching compared to abGCs from WT mice, while SCH58261 treatment alleviated this decrease in abGCs from APP/PS1 (*p* < 0.01 and *p* < 0.05, Fig. 6B). Moreover, abGCs from APP/PS1 mice exhibited a significant reduction in total dendritic length compared to WT mice (*p* < 0.01, Fig. 6C), while SCH58261 treatment partially reversed the total dendritic length of abGCs from APP/PS1 mice (*p* < 0.05, Fig. 6C). The effect of SCH58261 on dendritic spine alteration was further evaluated. The bottom of Fig. 6A showed representative images of dendritic fragments from the four groups. ANOVA analysis revealed a significant reduction in dendritic spines in the abGCs of APP/PS1 mice compared to WT mice (*p* < 0.001, Fig. 6D), while SCH58261 treatment abrogated the difference in dendritic spines between genotypes (*p* < 0.001, Fig. 6D). These results indicated that A_2A_R blockade significantly ameliorated dendritic development of abGCs in APP/PS1 mice.

### A_2A_R blockade ameliorated the LTP impairment of abGCs in APP/PS1 mice

**Fig. 7:**
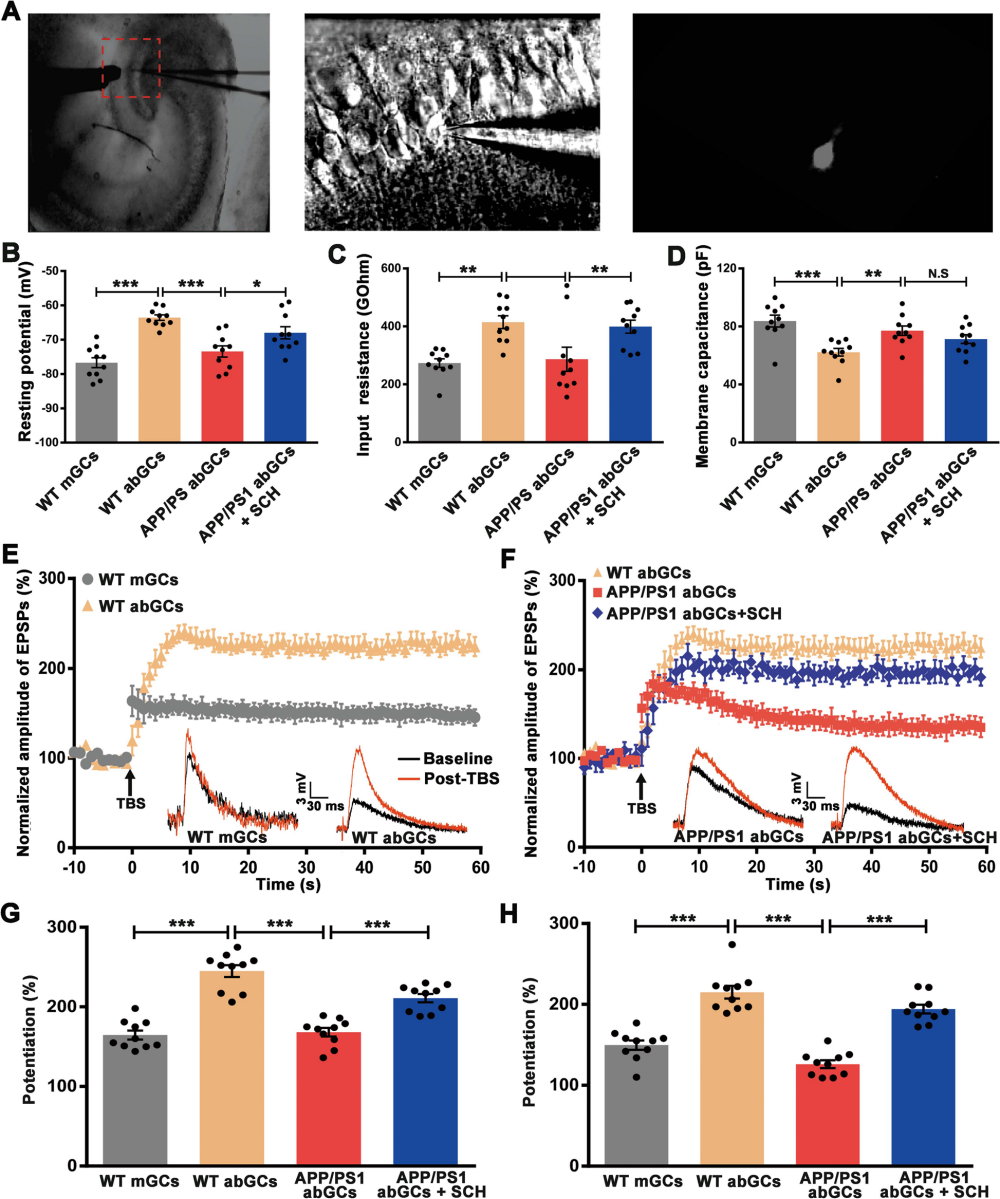
Blockade of A_2A_R Ameliorated Synaptic Plasticity Impairment of abGCs in APP/PS1 Mice. **A** Whole-cell recording from young abGCs. The left panel shows the low magnification IR-DIC view, with the stimulating electrode placed in the molecular layer to target the perforant path axons originating from the entorhinal cortex. The example picture depicts an abGC recorded with 40 × DIC (middle) and fluorescent view (right). **B**-**D** Intrinsic properties of abGCs and mGCs. Resting potential, input resistance, and membrane capacitance were compared between the groups. AbGCs from APP/PS1 mice displayed a more mature phenotype, which was partially reversed by SCH58261 treatment. **E** Summary of experiments showing LTP recorded in mGCs and abGCs of WT mice. The bottom row represents representative EPSPs taken before (black) and 50 min (red) after LTP induction by a physiologically relevant TBS (arrow). Scale bar = 30 ms and 3 mV. **F** Summary of experiments showing LTP recorded from abGCs of WT mice, APP/PS1 mice, and SCH58261-treated APP/PS1 mice. **G**-**H** Histograms showing the average amplitude of EPSPs during the first 10 min (**G**) and the last 10 min (**H**) post-TBS. AbGCs exhibited enhanced synaptic plasticity compared to mGCs in WT mice. The LTP amplitude decreased in abGCs of APP/PS1 mice, while treatment with SCH58261 eliminated the genotype-specific effects. Data are presented as the mean ± SEM, *n* = 10 neurons per group, with each neuron from an individual animal, *p < 0.05, **p < 0.01, ***p < 0.001 by one-way ANOVA followed by Sidak's post-hoc test

To investigate the effects of A_2A_R blockade on the synaptic functional properties of abGCs, we performed whole-cell patch-clamp recordings of granule cells in hippocampal slices prepared 1.5 month after retrovirus infection. At this time point, abGCs were functionally integrated into the hippocampal circuitry [41]. Newborn GFP + neurons in the inner granule cell layer were selected as abGCs (Fig. 7A), and GFP- cells localized in the same layer were selected as mGCs and were further confirmed by membrane properties. The intrinsic properties were measured: resting potential (V_rest_), input resistance (R_in_), and membrane capacitance (C_m_). In WT mice, 1.5 mpi abGCs exhibited higher V_rest_ and R_in_, and lower C_m_ compared to mGCs (*p* < 0.001, *p* < 0.01, and *p* < 0.001, Fig. 7B-D). However, abGCs in APP/PS1 mice showed a significant decrease in their V_rest_ and R_in_, and an increase in C_m_ compared with abGCs in WT mice (*p* < 0.001, *p* < 0.01, and *p* < 0.01, Fig. 7B-D), indicating accelerated maturation of abGCs in APP/PS1 mice. Treatment with SCH58261 eliminated genotype-specific effects on V_rest_ and R_in_ (*p* < 0.05 and *p* < 0.01, Fig. 7B-C), while had no significant effect on Cm (*p* > 0.05, Fig. 7D).

To evaluate synaptic plasticity, we recorded excitatory postsynaptic potentials (EPSPs) in response to stimulation of the medial perforant pathway. A theta burst stimulation was used to evoke LTP of granule cells. The EPSP amplitude was normalized with the average EPSP amplitude of the baseline. Significant LTP of EPSPs was reliably induced with TBS in 1.5 mpi abGCs of WT mice (Fig. 7E). The average EPSP amplitude during the first 10 min and the last 10 min after TBS for abGCs was significantly larger than that of mGCs (the first 10 min: mGCs 164.60 ± 5.60% vs. abGCs 245.00 ± 7.45%, *p* < 0.001, Fig. 7G; the last 10 min: mGCs 149.70 ± 5.83% vs. abGCs 215.00 ± 7.87%, *p* < 0.001, Fig. 7H), suggesting that 1.5 mpi abGCs exhibit enhanced synaptic plasticity compared to mGCs in WT mice. LTP amplitude of abGCs showed an effect of genotype and drug (Fig. 7E, F). LTP amplitudes of abGCs were decreased in APP/PS1 compared to WT mice (the first 10 min: 168.10 ± 5.40%, *p* < 0.001, Fig. 7G; the last 10 min: 126.10 ± 4.74%, *p* < 0.001, Fig. 7H), suggesting impaired LTP induction and maintenance. SCH58261 treatment recovered LTP amplitudes of abGCs in APP/PS1 mice (the first 10 min: 210.90 ± 5.23%, *p* < 0.001, Fig. 7G; the last 10 min: 194.20 ± 5.38%, *p* < 0.001, Fig. 7H). These results demonstrated that A_2A_R blockade ameliorated the LTP impairment of abGCs in APP/PS1 mice.

### A_2A_R blockade remodel subunit composition of NMDA receptors in abGCs of APP/PS1 mice

**Fig. 8:**
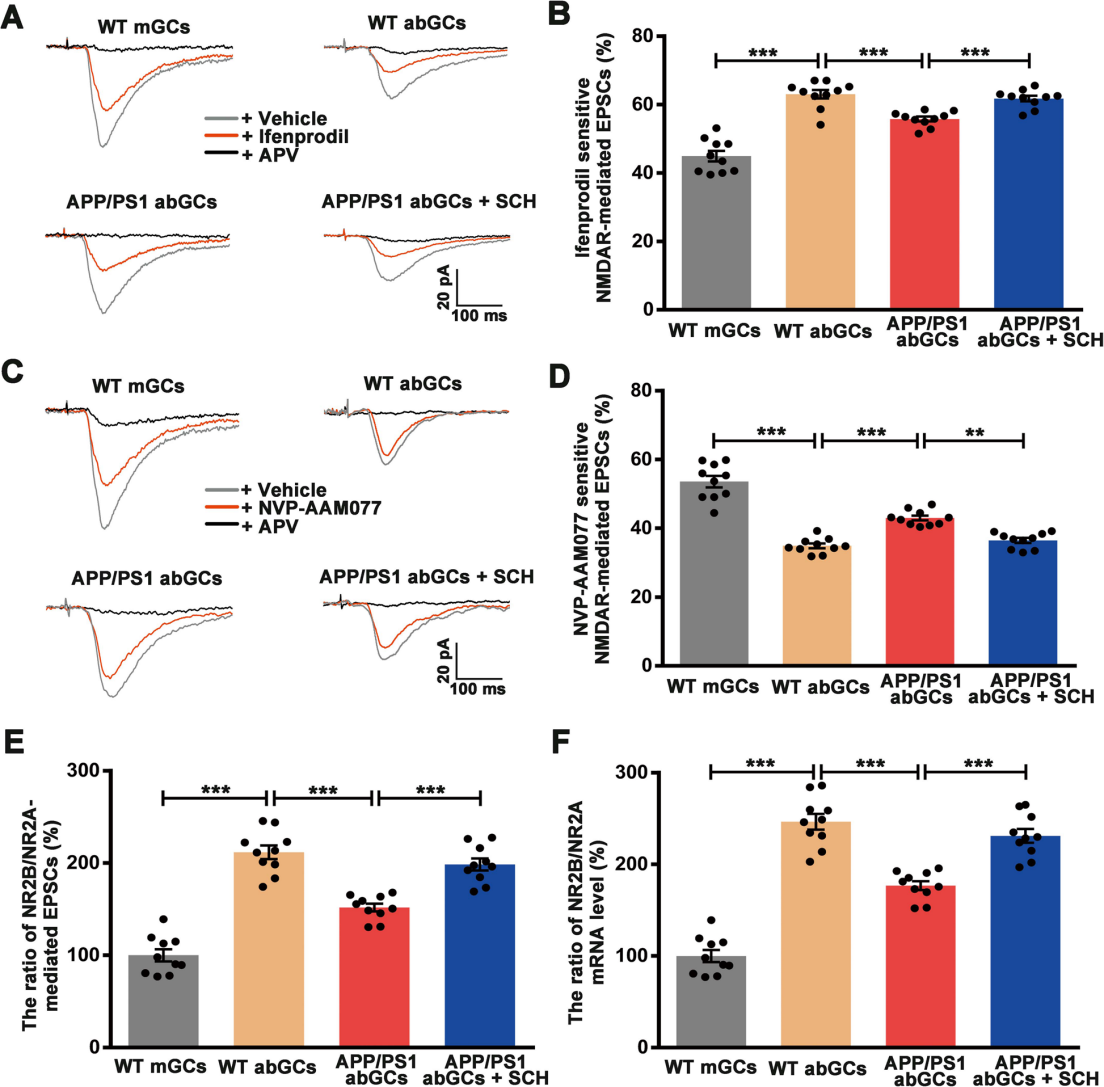
Blockade of A_2A_R Remodeled NMDA Receptors in abGCs of APP/PS1 Mice. **A** Pharmacologically isolated NMDAR-mediated EPSCs from granule cells recorded before (gray), after the application of ifenprodil (red), and followed by APV (black). Scale bar = 100 ms and 20 pA. **B** Summary of the inhibition of NMDAR-mediated EPSCs by ifenprodil, indicating the contribution of NR2B-containing NMDARs to the total NMDAR-mediated EPSCs in abGCs and mGCs. **C** Pharmacologically isolated NMDAR-mediated EPSCs from granule cells recorded before (gray), after the application of NVP-AAM077 (red), and followed by APV (black). Scale bar = 100 ms and 20 pA. **D** Summary of the inhibition of NMDAR-mediated EPSCs by NVP-AAM077, indicating the contribution of NR2A-containing NMDARs to the total NMDAR-mediated EPSCs in abGCs and mGCs. **E** Histograms showing the ratio between NR2B-mediated EPSCs and NR2A-mediated EPSCs. Treatment with SCH58261 reversed the decrease in the ratio observed in abGCs from APP/PS1 mice. **F** Histograms showing the relative expression of NR2B and NR2A mRNA in 1.5 mpi abGCs or mGCs. Treatment with SCH58261 reversed the decrease in the NR2B / NR2A mRNA ratio observed in abGCs from APP/PS1 mice. Data are presented as the mean ± SEM, *n* = 10 neurons per group, with each neuron from an individual animal, **p < 0.01, ***p < 0.001 by one-way ANOVA followed by Sidak’s post-hoc test

Enhanced plasticity in abGCs depends on the developmentally regulated synaptic expression of NR2B-containing NMDA receptors [11]. The synaptic plasticity alternation of abGCs in APP/PS1 mice indicates the changing of NR2B-containing NMDA receptors. During neuronal development, the NR2B subunit predominates, gradually being replaced by NR2A [42]. The synaptic plasticity alternation of abGCs in APP/PS1 mice indicates the changing of NR2B-containing NMDA receptors. Therefore, the effect of A_2A_R blockade on subunit composition of NMDA receptors in abGCs of APP/PS1 mice was next determined. We recorded NMDA-mediated EPSCs of GCs in the presence of AMPA and GABA receptor antagonists and confirmed this by the application of APV. The contribution of NR2B-containing NMDARs to NMDAR-mediated EPSCs in abGCs was examined using 3 μM fenprodil (Fig. 8A). Application of ifenprodil reduced the NMDAR-mediated EPSCs by 44.95 ± 4.88% and 63.03 ± 3.96% in mGCs and abGCs from WT mice, respectively (*p* < 0.001, Fig. 8B), suggesting higher NR2B levels in abGCs compared to mGCs. In contrast, the same treatment resulted in a 55.79 ± 2.23% and 61.77 ± 2.6% reduction in abGCs from APP/PS1 mice and SCH58261-treated APP/PS1 mice, respectively (*p* < 0.001; Fig. 8B). These results indicated a decrease in NR2B-containing NMDARs in abGCs of APP/PS1 mice, which was reversed by SCH58261 treatment. Similarly, the NR2A-mediated EPSCs were calculated by the change in EPSC amplitude after the application of NVP-AAM077 (Fig. 8C). The percentage of NR2A-mediated EPSCs to NMDA -mediated EPSCs in abGCs from WT mice was significantly lower than that in mGCs (*p* < 0.001, Fig. 8D). AbGCs from APP/PS1 mice showed an increased percentage of NR2A-mediated EPSCs compared to abGCs from WT mice (*p* < 0.001, Fig. 8D), while SCH58261 treatment reversed the increase (*p* < 0.01, Fig. 8D). Next, the ratio between the NR2B-containing NMDARs and NR2A-containing NMDARs (NR2B / NR2A) was evaluated to confirm the change in NR2B. In agreement with a previous study [11], NR2B / NR2A was significantly higher in abGCs from WT mice with respect to mGCs (211.74 ± 23.73%, *p* < 0.001, Fig. 8E). However, a significant reduction in the NR2B / NR2A was observed in abGCs from APP/PS1 mice compared to abGCs from WT mice (155.86 ± 13.27%, *p* < 0.001, Fig. 8E). SCH58261 treatment eliminated genotype-specific effects (198.49 ± 20.22%, *p* < 0.001, Fig. 8E), suggesting that A_2A_R blockade prevents the decrease in the NR2B / NR2A ratio in abGCs of APP/PS1 mice. It was reported that NR2A mRNA expression increases in association with the downregulation of NR2B mRNA expression during neuron maturation [42]. Using single-cell real-time PCR, we further evaluated the expression of NR2B / NR2A in abGCs and found that the NR2B / NR2A mRNA relative expression of abGCs was distinctly higher than that of mGCs in WT mice (*p* < 0.001, Fig. 8F). A significant reduction in the NR2B / NR2A was observed in abGCs from APP/PS1 mice compared to abGCs from WT mice (*p* < 0.001, Fig. 8F), while SCH58261 treatment eliminated genotype-specific effects (*p* < 0.001, Fig. 8F), indicating that blockade of A_2A_R remodeled NMDAR receptors and increased the proportion of NR2B receptors in abGCs from APP/PS1 mice. These results suggest that the modulation of subunit composition of NMDA receptors by A_2A_R may play a role in synaptic plasticity of abGCs.

## Discussion

### Neuroprotective effects of A_2A_R blockade in AD

Enhanced activation of A_2A_R is considered a plausible pathophysiological mechanism in brain of the mouse model for AD and in the human AD brain [19–21]. Selective pharmacological or genetic A_2A_R inhibition improves learning and memory in AD models, suggesting that A_2A_R may be a possible therapeutic target in AD [43]. However, the mechanisms responsible for the protective effects of A_2A_R blockade on cognitive impairment in AD remain unclear. Previous studies have primarily focused on various pathological components of AD, such as Aβ peptide deposits, tau hyperphosphorylation, neuroinflammation, neuronal injury, and neurotransmitter homeostasis [44–47]. Regular consumption of caffeine, a non-selective A_2A_R antagonist, protected transgenic mouse models of AD against cognitive impairment, while also mitigating amyloid and tau lesions, neuroinflammation, and oxidative stress in their brains [47, 48]. Chronic treatment with the selective A_2A_R antagonist MSX-3 has been found to prevent memory disorders and decrease Aβ peptide deposits in APP/PS1 mice [44]. Selective A_2A_R antagonist SCH58261 prevents memory dysfunction and synaptotoxicity caused by beta-amyloid peptides via the p38 mitogen-activated protein kinase pathway [46]. Furthermore, genetic deletion of the A_2A_R has been found to improve spatial memory deficits and plasticity while reducing hippocampal neuroinflammation and tau hyperphosphorylation in a model of tauopathy [45]. A_2A_Rs have also been significantly upregulated by astrocytes in the brains of AD patients, as well as in mice with amyloid lesions, while conditional ablation of astrocytic A_2A_R alleviated memory deficits in aging hAPP mice [20]. Glutamate uptake was found to be reinstated in Aβ-treated astrocytes through A_2A_R blockade [49].

Adult neurogenesis in the hippocampus plays a crucial role in various cognitive functions, such as memory and pattern separation [9]. In mouse models of AD, neurogenesis is impaired, and the generated granule neurons fail to integrate into existing networks. Genetic destruction of neurogenesis in APP/PS1 mice can aggravate DG hyperactivity and spatial memory impairment [50]. On the contrary, pharmacological stimulation of AHN improves cognition in AD mice [51]. Similar to rodents, there is evidence indicating a decline in hippocampal neurogenesis during aging in humans [52]. Clinical evidence also shows that drugs promoting hippocampal neurogenesis can significantly improve spatial memory in MCI patients [53]. Targeted neurogenesis pathway-based gene analysis identifies A_2A_R associated with hippocampal volume in MCI and AD. However, no previous studies have reported whether A_2A_R blockade can improve spatial memory in AD through protecting synaptic plasticity and functional integration of adult-born neurons during their maturation phase when they contribute to memory processes. In our present study, we found that the selective A_2A_R antagonist SCH58261 can protect the synaptic plasticity of abGCs by recovering dendritic morphology and NR2B level of abGCs in APP/PS1 mice. Recovered plasticity of abGCs ameliorates synaptic plasticity and network activity in the DG, ultimately reversing early spatial memory deficits in the APP/PS1 mouse model of AD. Our present study uncovered previously unsuspected mechanisms underlying the protective effects of A_2A_R blockade on cognitive impairment in AD. These data support the notion that A_2A_R blockade is of therapeutic value for AD.

### Adenosine A_2A_ receptor and adult hippocampal neurogenesis

The adenosine A_2A_ receptor is a crucial modulator of the nervous system, influencing various physiological functions, such as cognitive function and memory. In a physiological state, A_2A_R activation promotes adult hippocampal neurogenesis. In the DG of wild type rat, A_2A_R activation promoted neural stem cell self-renewal, protected committed neuronal cells from cell death and contributed to a higher density of immature and mature neuronal cells [26]. Animal models of spinal cord injury have demonstrated that activation of A_2A_R enhances neurogenesis and reduces neuronal damage [54]. Conversely, A_2A_R knockout mice show cognitive impairment due to reduced neuronal proliferation and abnormal changes in the expression of synaptic proteins in the hippocampus [55]. Recent research has revealed that noise exposure damages cognitive function in adult mice by decreasing the number of newborn neurons in the hippocampus. The A_2A_R agonist CGS21680 can effectively increase the number of newborn neurons in the adult hippocampus, mitigating hearing and cognitive function damage caused by noise exposure [56]. A_2A_R activation may enhance adult neurogenesis in a physiological state, while abnormal elevation of A_2A_R level in a pathological state induces adult neurogenesis impairment [22, 57, 58]. Cisplatin treatment has been found to elevate the expression of A_2A_R and induce impairments in neural progenitor proliferation and dendrite morphogenesis of adult-born neurons. A_2A_R inhibition by the antagonist KW-6002 prevented cisplatin-induced impairments in neurogenesis and cognitive function [58]. On the other hand, a recent study revealed that inhibition of A_2A_R induced impulsive behavior accompanied by increased immature neuroblast proliferation in the hippocampus [59].

Here, we found that the APP/PS1 mouse model of AD showed upregulation of adenosine A_2A_R, hampering the synaptic plasticity of newborn neurons. A_2A_R inhibition prevented impairments in dendrite morphogenesis and synaptic plasticity of adult-born neurons, ultimately improving DG-related memory in APP/PS1 mice. In conclusion, both previous experimental evidence and our present results suggest that A_2A_R at a physiological level can promote neurogenesis and protect memory. Abnormal levels of adenosine, either too low or too high, in various pathological conditions can cause neurogenesis abnormality, resulting in memory impairment. Further studies are needed to investigate the mechanism by which A_2A_R regulates neurogenesis.

### NR2B-dependent plasticity of adult born granule cells

Synaptic plasticity of the DG is fundamental for DG-related memory function and is modulated by neurogenesis in the adult hippocampus [14]. Ablation of adult hippocampal neurogenesis by X-ray irradiation results in a significant reduction in the amplitude of DG excitatory postsynaptic potentials and population spike evoked by perforant pathway stimulation [60]. Deficits in DG synaptic plasticity are rescued when neurogenesis is restored [61]. Immature abGCs aged 4–6 weeks exhibit greater synaptic plasticity compared to mature granule cells, with increased LTP amplitude and decreased LTP induction threshold [11]. Electrophysiology of hippocampal slices from mice with selective deletion of the NR2B subunit in abGCs revealed that NR2B receptors are key mediators in enhancing synaptic plasticity in abGCs. Deletion of NR2B receptors impairs LTP in the DG and reduces the dendritic complexity of abGCs, and tetanic stimulation fails to induce LTP in abGCs lacking NR2B [62]. These findings highlight the significance of the developmentally regulated synaptic expression of NR2B receptors in promoting enhanced plasticity in abGCs.

NMDA receptors, membrane-bound ionotropic glutamate receptors, are crucial for synaptic plasticity, particularly in the hippocampus. NMDARs consist of two NR1 subunits and two NR2 subunits. Among the four described NR2 subtypes (NR2A, NR2B, NR2C, and NR2D), NR2A and NR2B can form complexes with NR1 [63]. In the hippocampus, the expression level of NR2 subunits dynamically changes during postnatal development. Application of a specific NR2B antagonist results in a 72% reduction of excitatory postsynaptic currents (EPSCs) in abGCs and only a 25% reduction in mGCs, suggesting that the NR2B receptor is the predominant NR2 subtype in 4–6 weeks and is associated with enhanced synaptic plasticity of abGCs [11]. After the critical period of 4 to 6 weeks, NR2B receptors are gradually replaced by NR2A receptors, which become the predominant NR2 subunit in mGCs [64]. In the present study, we observed an accelerated shift in synaptic NMDA receptor subtypes from NR2B to NR2A in APP/PS1 mice, which was accompanied by a significant decrease in LTP of abGCs. These results suggest that AD accelerates the maturation of newborn DG cells by promoting a developmental switch in synaptic NMDAR subtypes, leading to the acquisition of a mature LTP phenotype.

### Adenosine A_2A_ receptor modulate NMDA receptor expression patterns

Adult neurogenesis has been implicated in the spatial memory of the DG, and understanding how A_2A_R affects the maturation of newborn DG cells is critical for comprehending the protective effects of A_2A_R inhibition on early deficits in memory and synaptic plasticity in AD. The NR2B subunit plays a vital role in the synaptic plasticity of abGCs [10, 11]. Previous studies have demonstrated that the activation of A_2A_Rs can regulate NMDA receptor activation and consistently facilitate NMDAR currents [65]. The interaction between NMDA and A_2A_R can form a compound, and the amount of the compound is markedly higher in hippocampal cells from APP/PS1 model mice than from WT mice. In our present study, we found that the impairment of LTP in abGCs from APP/PS1 mice was accompanied by a significant reduction in the NR2B / NR2A ratio at the immature synapse of abGCs. Interestingly, treatment with the A_2A_R antagonist SCH58261 rescued synaptic plasticity deficits and the reduction in the NR2B / NR2A ratio in abGCs from APP/PS1 mice. Our findings are consistent with a previous study showing that A_2A_R blockade remodeled striatal NMDA receptors in Huntington’s disease mice [66]. More generally, our study confirms that A_2A_R is altered in AD and that this alteration has an impact on the synaptic maturation of newborn DG cells.

Metabotropic glutamate 5 receptors (mGluR5) might be candidates for the ability of A_2A_R to regulate NMDA receptors in AD. In fact, A_2A_R and mGluR5 are coexpressed and functionally interactive, and A_2A_R controls the ability of mGluR5 receptors to enhance the response of NMDA receptors [67]. Activation of A_2A_R enables the coactivation of mGluR5 and NMDAR in the hippocampus, leading to robust phosphorylation of NR2B (Tyr1472) receptors. This phosphorylation anchors NR2B receptors on postsynaptic membranes, preventing their internalization. Another candidate mediating the interaction between A_2A_R and NMDA receptors is the dopamine D1 receptor. A recent study showed that dopamine D1 receptor-evoked NR2B receptor phosphorylation in the hippocampus is also regulated by endogenous adenosine and A_2A_R [68]. In addition, brain-derived neurotrophic factor (BDNF) is an important trophic factor that regulates synaptic transmission and modulates NMDA receptor activity through presynaptic and postsynaptic receptors. Endogenous activation of A_2A_R is essential for BDNF-mediated regulation of NMDA receptors [26].

In healthy human brains, endogenous adenosine acting on A_2A_R potentiates the effects of NR2B receptors. However, we found that blockade of A_2A_R increased the NR2B / NR2A ratio in abGCs from APP/PS1 mice. The differential effects of A_2A_R on NR2B regulation observed in WT and APP/PS1 mice may be due to altered signaling pathways in the hippocampus. Aberrant mGluR5 signaling and associated synaptic failure are considered emerging pathophysiological mechanisms of AD. Reduced mGluR5 activity has been reported in both animal models of AD and AD patients [69]. Functional alteration of the dopamine D1 receptor in hippocampal cell membranes in AD has also been reported [70] Changes in BDNF levels have been reported in both animal models of AD and AD patients [71]. Therefore, changes in A_2A_R signal transduction likely occur in AD models, resulting in alterations in NMDA receptor expression patterns. Further experiments are needed to explore the underlying molecular mechanisms.

## Conclusions

In summary, our study indicated that the selective A_2A_R antagonist SCH58261 significantly improved spatial cognitive deficits by restoring LTP and rebalancing network excitation/inhibition in the DG region during the early stages of AD. Furthermore, treatment with SCH58261 alleviated alterations in dendritic morphology and impaired synaptic plasticity of abGCs by modulating subunit composition of NMDA receptors and increasing NR2B expression in abGCs in APP/PS1 mice. These findings underscore the potential of A_2A_R blockade as a neuroprotective intervention for the treatment of AD, as it targets early spatial memory impairments by reversing synaptic abnormalities in abGCs.

### Supplementary Information

**Additional file 1: Figure S1.** The effect of SCH58261 on the protein expression of A_2A_R.

## Data Availability

The datasets used and/or analyzed during the current study are available from the corresponding author on reasonable request.

## Abbreviations

A_2A_R: Adenosine A_2A_ receptors
AD: Alzheimer’s disease
LTP: Long-term potentiation
abGCs: Adult-born granule cells
mGCs: Mature granule cells
Aβ: Amyloid-β
DG: Dentate gyrus
MWM: Morris water maze
OLT: Novel object location recognition
fEPSPs: Field excitatory postsynaptic potentials
EPSPs: Excitatory postsynaptic potentials
