# Supplementary material for: Blockade of adenosine A2A receptors reverses early spatial memory defects in the APP/PS1 mouse model of Alzheimer’s disease by promoting synaptic plasticity of adult-born granule cells
Source: Alzheimers Res Ther. 2023 Oct 30;15:187. doi: 10.1186/s13195-023-01337-z (PMC10614339; doi:10.1186/s13195-023-01337-z)
Supplement: Supplementary file 1 — Additional file 1: Figure S1. The effect of SCH58261 on the protein expression of A2AR. [file 13195_2023_1337_MOESM1_ESM.doc]

**Figure 1**

**
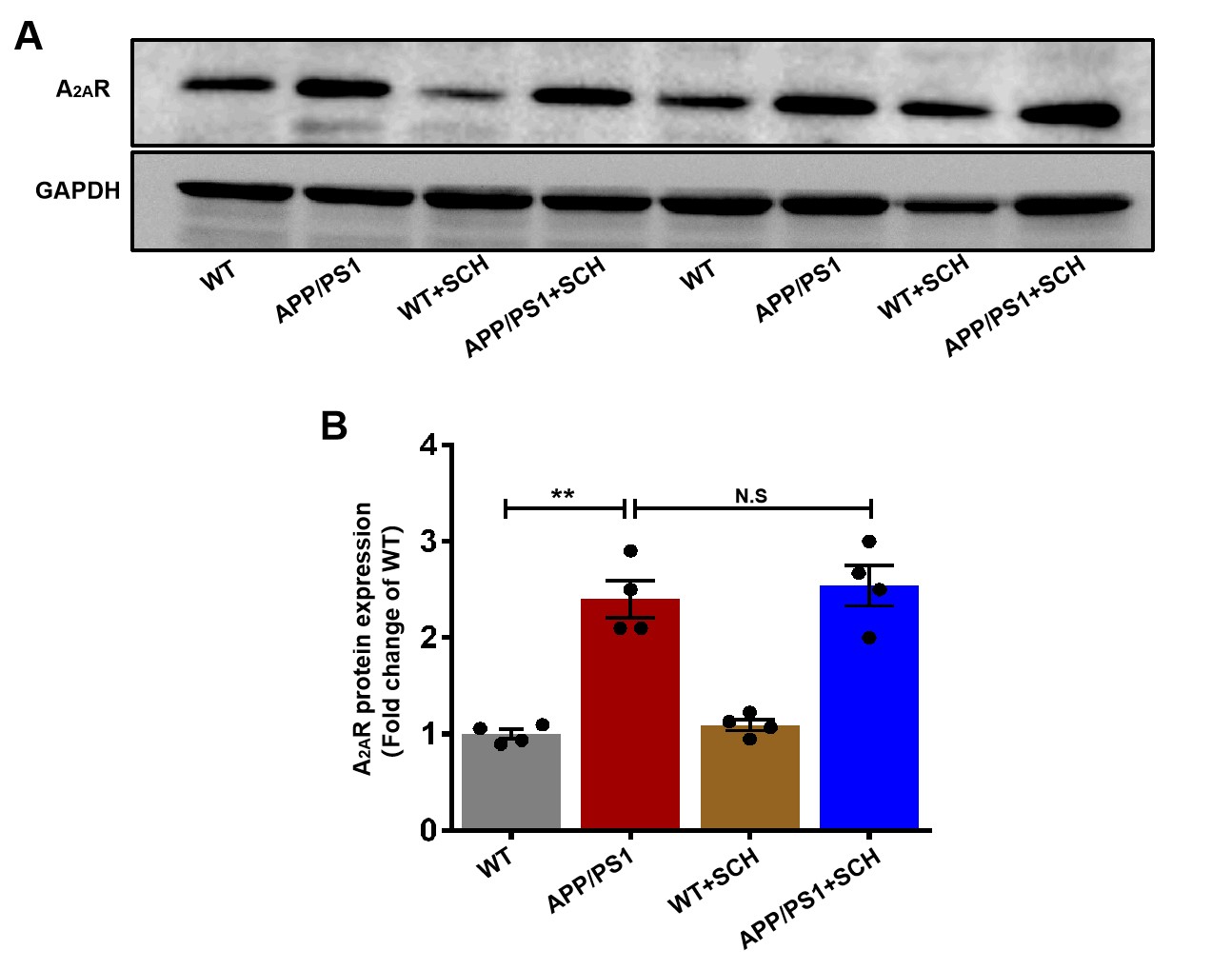
**

**Figure 1. The effect of SCH58261 on the protein expression of A_2A_R.** (A) Representative Western blots of A_2A_R in the hippocampus. (B) Histograms showing the significant increase of A_2A_R protein expression in APP/PS1 mice, whereas treatment with SCH58261 had no effect. Each value is represented as mean ± SEM. n = 4 animals per group, ***p < 0.01 vs. WT group using one-way ANOVA followed by Sidak’s post-hoc test.
